# Hierarchical, porous CuS microspheres integrated with carbon nanotubes for high-performance supercapacitors

**DOI:** 10.1038/srep16584

**Published:** 2015-11-16

**Authors:** Yang Lu, Xianming Liu, Weixiao Wang, Jinbing Cheng, Hailong Yan, Chengchun Tang, Jang-Kyo Kim, Yongsong Luo

**Affiliations:** 1 Key Laboratory of Advanced Micro/Nano Functional Materials, School of Physics and Electronic Engineering, Xinyang Normal University, Xinyang, P. R. China; 2 School of Material Science and Engineering, Hebei University of Technology, Tianjin, P. R. China; 3 College of Chemistry and Chemical Engineering, Luoyang Normal University, Luoyang, P. R. China; 4 Department of Mechanical and Aerospace Engineering, The Hong Kong University of Science and Technology, Clear Water Bay, Kowloon, Hong Kong, P. R. China

## Abstract

Carbon nanotubes (CNTs) incorporated porous 3-dimensional (3D) CuS microspheres have been successfully synthesized via a simple refluxing method assisted by PVP. The composites are composed of flower-shaped CuS secondary microspheres, which in turn are assembled with primary nanosheets of 15–30 nm in thickness and fully integrated with CNT. The composites possess a large specific surface area of 189.6 m^2^ g^−1^ and a high conductivity of 0.471 S cm^−1^. As electrode materials for supercapacitors, the nanocomposites show excellent cyclability and rate capability and deliver an average reversible capacitance as high as 1960 F g^−1^ at a current density of 10 mA cm^−2^ over 10000 cycles. The high electrochemical performance can be attributed to the synergistic effect of CNTs and the unique microstructure of CuS. The CNTs serve as not only a conductive agent to accelerate the transfer of electrons in the composites, but also as a buffer matrix to restrain the volume change and stabilize the electrode structure during the charge/discharge process. The porous structure of CuS also helps to stabilize the electrode structure and facilitates the transport for electrons.

The ever worsening energy and global warming issues call for the urgent development of clean and high performance energy storage devices. As a new class of energy storage devices, supercapacitors (SCs) have attracted great attention over the past decades owing to their prominent characteristics of high power density, long cyclic life and superior safety^1^,^2^,^3^,^4^,^5^,^6^. It is generally known that the capacitance of supercapacitors relies largely on the electrode material^6^. Three major types of electrode materials have thus far been considered for electrodes in SCs: namely, carbon materials^7^, metal oxides/hydroxides^8^ and conducting polymers^9^. Apart from these widely-used electrode materials, some transition-metal chalcogenides have received increasing attention as electrode materials in view of their high theoretic capacities, abundant availability and low costs^10^,^11^,^12^,^13^. Among several metal sulfide compounds, CuS is an important multifunctional semiconductor with potential applications in gas sensors, lithium ion batteries and solar energy devices^14^,^15^,^16^,^17^,^18^,^19^. For instance, various studies have been made to use CuS as a cathode material in rechargeable batteries with charge/discharge stability for up to 1000 cycles^20^ and as a counter electrode in photoelectrochemical cells owing to its promising redox reactions with polysulfide electrolyte^21^. To the best of our knowledge, however, very few studies have appeared on the application of CuS in SCs. Zhu *et al.* utilized the template-induced chemical conversion route to prepare CuS nanoneedles on carbon nanotubes as an electrode, which exhibited a specific capacitance of 122 F g^−1^ in KOH electrolyte^22^. More recently, Huang *et al.* formed CuS nanosheets as an electrode with a specific capacitance of 833 F g^−1^ using a solvothermal method^23^. Relatively low capacitances have so far been achieved from the above CuS systems, owing to small surface areas of the CuS electrodes and the poor interfacial kinetics for charge-transfer at the liquid/solid interface. Hence, developing new approaches to forming CuS-based supercapacitors with large surface areas and efficient interfacial kinetics is very desirable.

Combining different materials into hybrid nanostructures has attracted considerable interest^24^, and many reports have focused on assembling one-dimensional components with semiconductors or metal nanoparticles^25^,^26^. Owing to their unique tubular structure, high electrical and thermal conductivities and large surface areas, carbon nanotubes (CNT) have been considered as ideal additives to enhance the electrochemical characteristics of rechargeable batteries and SCs with much improved energy storage capacities^27^,^28^,^29^. Various methods, including covalent and noncovalent approaches, have been developed to assemble hybrid structures using CNT and semiconductor nanoparticles^30^. For both approaches, organic molecules are usually used to crosslink the nanoparticles and CNT together, but the incorporation of these organic molecules inevitably degrades the charge transfer between the two components^26^. Direct connections between them without the cross-linking molecules are desired to form effective hybrid junctions at the interfaces^31^. *In situ* synthesis of CNT/nanoparticle hybrids will make it possible to form direct connections, so as to facilitate charge interactions and provide a driving force for separating charge carriers^32^.

Taking into account all the aforementioned issues together, we herein develop a novel architecture consisting of 3D hierarchical CuS microspheres integrated with CNT, via a simple reflux route. The CNT are fully embedded and intermingled with the CuS nanosheets on one end, while they are exposed outside the microspheres on the other to form 3D conducting networks between the whole active materials. The CNT within the unique hierarchical structure not only enhances the electronic conductivity of the electrode, but also functions as an elastic buffer for releasing the strain of CuS particles during the charge/discharge process. As an anode material for supercapacitors, the CuS/CNT composites combine the advantages of high capacitance of the active primary nanostructure and easy handling of microscale secondary particles.

## Results and Discussion

### Structures and morphologies

**Figure 1:**
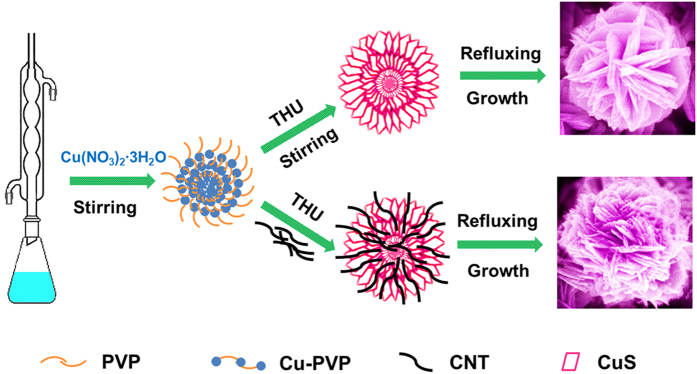
Schematic diagram of the fabrication process for the CuS/CNT composites.

**Figure 2:**
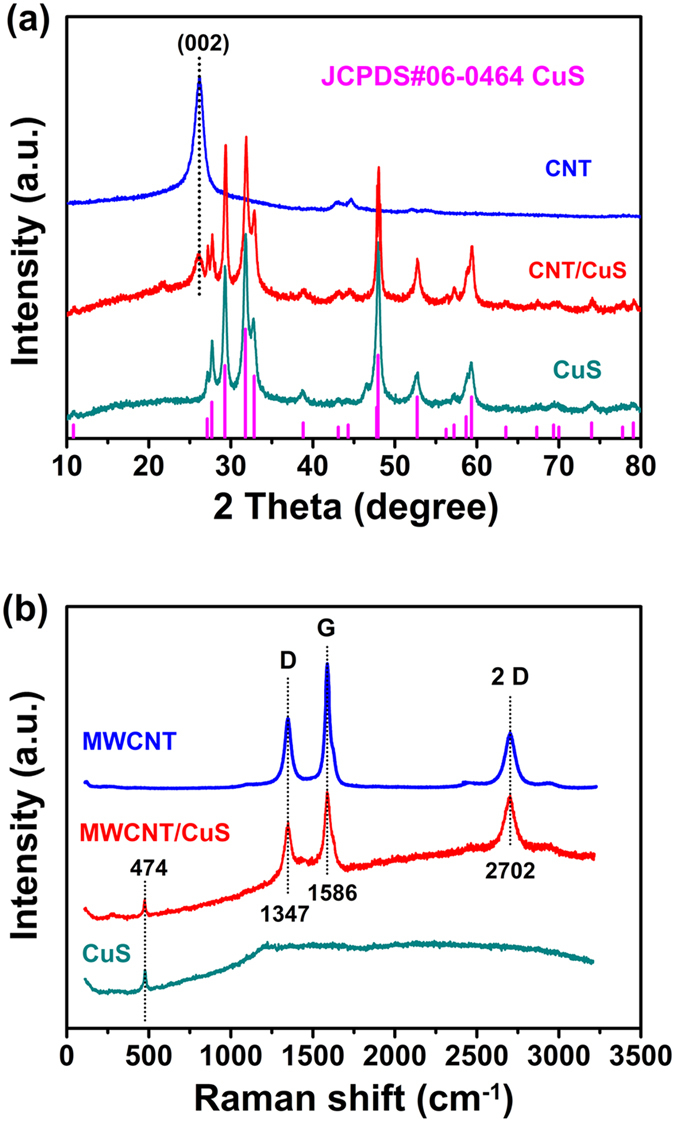
XRD patterns (**a**) and Raman spectra (**b**) of CuS, CNT and CuS/CNT composites.

Figure 1 illustrates the synthesis procedure of the CuS/CNT composites, where the PVP only served as a template for the formation of the 3D flower-like microspheres. THU serves as a sulfur source, which combines with Cu^2+^ and EG to form [Cu(Tu)m(EG)n]^2+^ 33,34. This process greatly slowed the formation of CuS and led to its anisotropic growth. Consequently, CuS nanosheets self-assembled to the hierarchical flower-like microspheres. The X-ray diffraction patterns given in Fig. 2a show that the peaks of CuS and CuS/CNT were sharp indicating their good crystallinity. The crystalline peaks appeared at 10.8^o^ 2θ, 27.7^o^ 2θ, 29.2^o^ 2θ, 31.7^o^ 2θ, 32.7^o^ 2θ, 38.8^o^ 2θ, 48^o^ 2θ, 52.5^o^ 2θ and 59.2^o^ 2θ, corresponding to the (002), (100), (101), (102), (103), (105), (107), (108) and (203) planes of CuS (JCPDS card no. 06–0464), respectively. The peaks for CNT at 25.9^o^ 2θ (002) and 42.8^o^ 2θ (100) attribute to the hexagonal graphite structure, indicating high electrical conductivities. The diffraction peaks of CuS remained much the same even after the incorporation of CNT, a reflection of little change of the phase structure, and the crystallite size calculated using the Scherrer formula of full profile fitting was 23 nm.

The structural features and the possible formation mechanism of CuS/CNT composites were studied using the Raman spectroscopy, as shown in Fig. 2b. Three prominent peaks at 1586 cm^−1^ (G band), 1347 cm^−1^ (D band) and 2702 cm^−1^ (2D band) were observed for the pristine CNT. They originate from the Raman-active in-plane atomic displacement E_2g_ mode of the tangential C-C stretching vibrations (both longitudinally and transversally on the nanotube axis), disorder-induced features of the CNT and the overtone of D band^35^. The spectral intensities of the D, G and 2D bands were slightly modified while their positions remained unaltered from the pristine CNT. Apart from the three peaks for CNT, another Raman band appeared at 474 cm^−1^ for the CuS/CNT composites, attributed to pristine CuS^36^. The intensity ratio (ID/IG) of D-band to G-band for the CuS/CNT composites is 0.85, which is higher than the pure CNT (0.79). The enhancement of the D band is due to the defects and vacancies introduced by acid treatment of CNT and the formation of CuS. In addition, FWHM values of the G-band present similar trend: 64 cm^−1^ (CuS/CNT) > 45 cm^−1^ (CNT), which also demonstrates the presence of CuS. The above observations agree well with the XRD results indicating that CNT were embedded into the CuS particles, an important attribute for efficient transfer of ions and electrons during cycles^37^.

**Figure 3:**
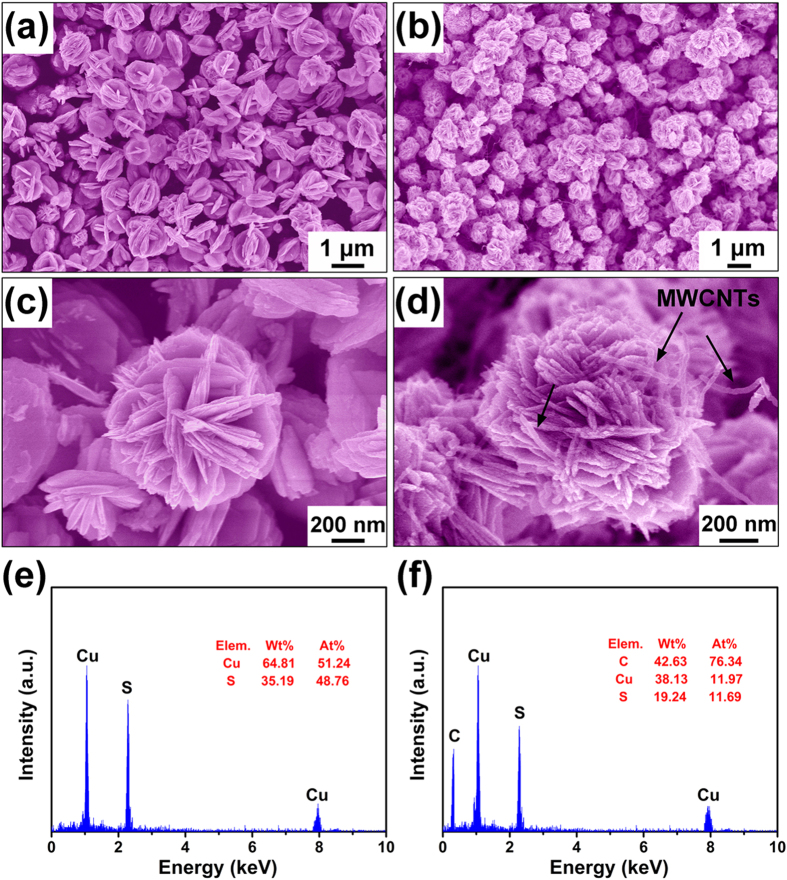
SEM images of CuS (**a,c**), CuS/CNT composites (**b,d**); and EDX patterns of CuS (**e**), CuS/CNT composites (**f**).

The SEM images of CuS and CuS/CNT composites along with their energy dispersive X-ray spectra are given in Fig. 3. The neat CuS particles were in the form of peony flower-like microspheres of ~ 1 μm in diameter. Each microsphere was composed of new tens of bundled nanosheets of 15–30 nm in thickness (Fig. 3a,c). The CuS/CNT composites maintained much the same shape and size of the CuS microspheres, with the exception of the integrated CNT (Fig. 3b,d). The well dispersed CNT are seen fully embedded into the CuS microspheres (Fig. 3d). The EDX spectra shown in Fig. 3e and f confirmed that both S and C coexists along with Cu in CuS/CNT composites. The molar ratio of Cu to S was approximately 1:1, agreeing well with the stoichiometry of CuS.

**Figure 4:**
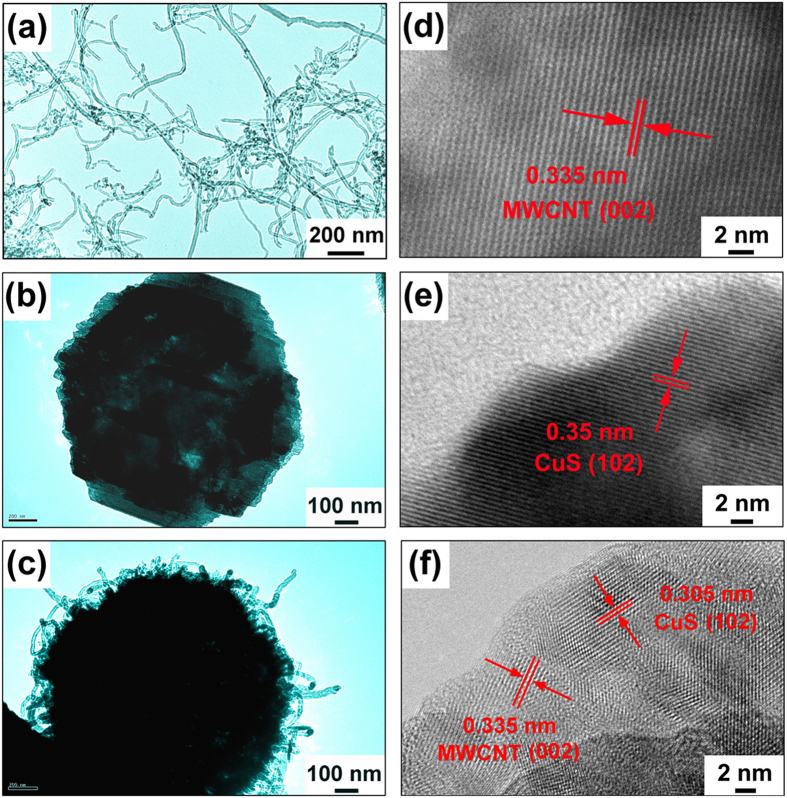
TEM images of CNT (**a,d**), CuS (**b,e**) and CuS/CNT composites (**c,f**): (**a–c**) low magnification, and (**d–f**) HRTEM images.

The TEM image presents well-dispersed CNT (Fig. 4a) and parallel lattice fringes with a basal distance of 0.335 nm (Fig. 4d), consistent with the (002) plane of CNT. The individual flower-like CuS microspheres were an assembly of a number of nanosheets (Fig. 4b) and the parallel lattice fringes with a distance of 0.305 nm (Fig. 4e) is consistent with the (102) plane of CuS. The CuS/CNT composites exhibited a number of individual and entangled CNT on the surface of the CuS microspheres, as shown in Fig. 4c. It appears that many CNT were embedded between the petal-look nanosheets inside the microspheres on one end, while they were exposed to the surface on the other. Actually the conductivity of CuS increases from generally 10^−3^ S cm^−1^ to 0.471 S cm^−1^ in our case (measured by standard four-probe method) by incorporating with CNT. The d-spacings measured from the lattice fringes were 0.335 and 0.305 nm (Fig. 4f), corresponding to the (002) plane of CNT and the (102) plane of CuS, respectively.

**Figure 5:**
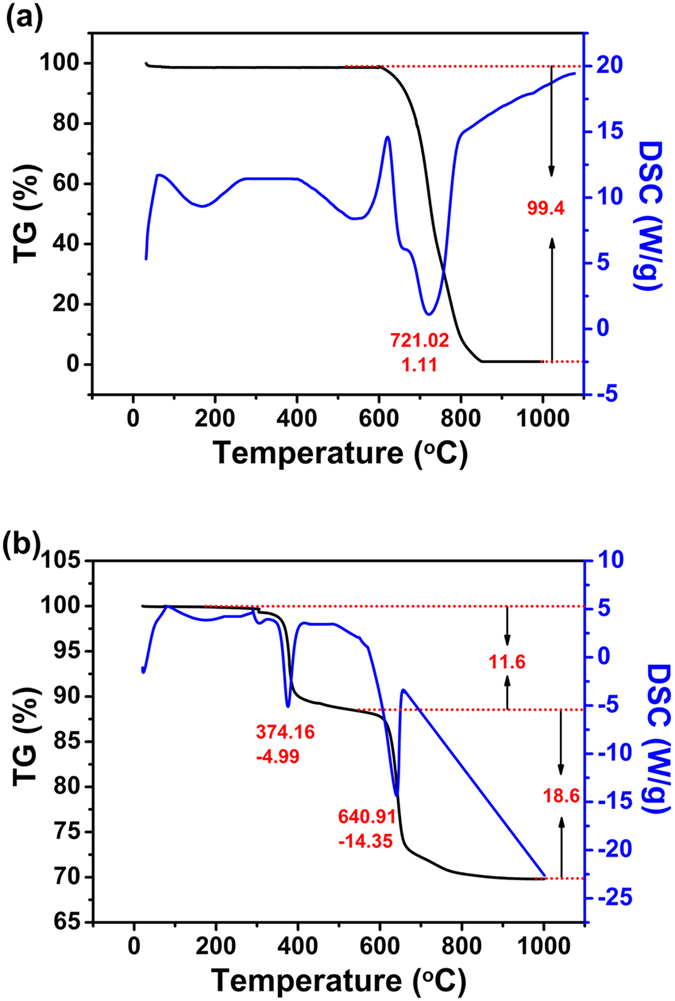
TG (black line) and DSC (blue line) curves for CNT (**a**), and CuS/CNT composites (**b**) in air at a heating rate of 10 ^o^C min^−1^.

The results obtained from the TG and DSC analyses are shown in Fig. 5. The oxidation of neat CNT into CO_2_ occurred between 600 and 850 ^o^C (Fig. 5a). In contrast, CuS/CNT composites exhibited two major weight losses in the TG curve along with two corresponding exothermic peaks in the DSC curve (Fig. 5b). The weight loss between 350 and 400 ^o^C was caused by the oxidation of CuS, while that between 600 and 850 ^o^C was attributed to the combustion of CNT. Therefore, the content of carbon in the CuS/CNT composites is estimated to be ca. 18.6%.

The nitrogen adsorption and desorption isotherm and pore size distribution of the CuS/CNT composites are shown in Figure S1. The isotherm is type IV with H3 hysteresis, demonstrating the presence of mesoporous. According to the isotherms, the BET surface area of the CuS/CNT composites was calculated to be 190 m^2^ g^−1^. Together with the pore distribution analysis (inset in Figure S1) by density functional theory (DFT), the CuS/CNT composites reveal mesoporous structures with the pore size confined at ~ 7 nm. Such flower-like hierarchical morphology with well-developed pore structures are advantageous for energy storage applications^8^.

### Electrochemical performance of SCs

**Figure 6:**
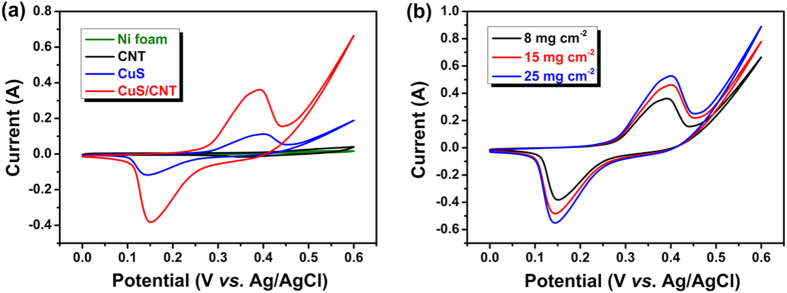
(**a**) CV curves of CuS, CNT, and CuS/CNT composites, and (**b**) mass-dependent CV curves of the CuS/CNT electrodes in 2 M KOH electrolyte at a 20 mV s^−1^ scan rate.

Figure 6a plots the CV curves of the electrodes made from CuS, CNT, CuS/CNT with a mass loading of 8 mg cm^−2^ and pure Ni foam measured at a 20 mV s^−1^ scan rate. The shapes of the CV prove that the capacitance characteristic is very different from that of the electric double-layer capacitance where the shape is generally close to an ideal rectangular shape. Several important features are noted as follow: (i) Redox peaks were present for the CuS and CuS/CNT electrodes which store electrical energy mainly by means of reversible faradaic redox reactions. This phenomenon may result from the CuS/CuSOH redox pair, basing on the following electrochemical reaction:^38^


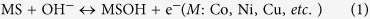


The redox reaction of the CuS electrode promotes charge storage and the transmission of the electrons and hydroxide ions in the electrode materials. (ii) The CV curve of the CuS/CNT composites showed a larger encircled area, suggesting a higher capacitance. (iii) Compared with the negligible C_s_ from the CNT and Ni foam, one can securely identify that the capacitance of the CuS/CNT composites mostly derives from the CuS microspheres rather than CNT or Ni foam.

Currently, the high performance of many materials with proven records on a laboratory scale may not be fully realized when they are scaled up upon commercialization. In particular, electrochemical energy storage devices typically experience performance degradation when tested with samples containing active materials of increased masses and thickness^39^. To demonstrate the capability of the current CuS/CNT electrodes for practical applications, the effect of electrode mass loading on specific capacitance was specifically investigated. Figure 6b presents mass-dependent CV curves of the CuS/CNT electrodes in the range from 8 to 25 mg cm^−2^ at 20 mV s^−1^. As the mass loading increases, the ion diffusion length in CuS/CNT composites also increases. Therefore, along with the mass loading increases, the C_s_ decreases due to the kinetics of ion transport in CuS/CNT composites with low ion diffusion rate. A maximum C_s_ of 2028 F g^−1^ was achieved for a loading of 8 mg cm^−2^. The specific capacitance decreased to 922 F g^−1^ when the loading increased to 25 mg cm^−2^.

**Figure 7:**
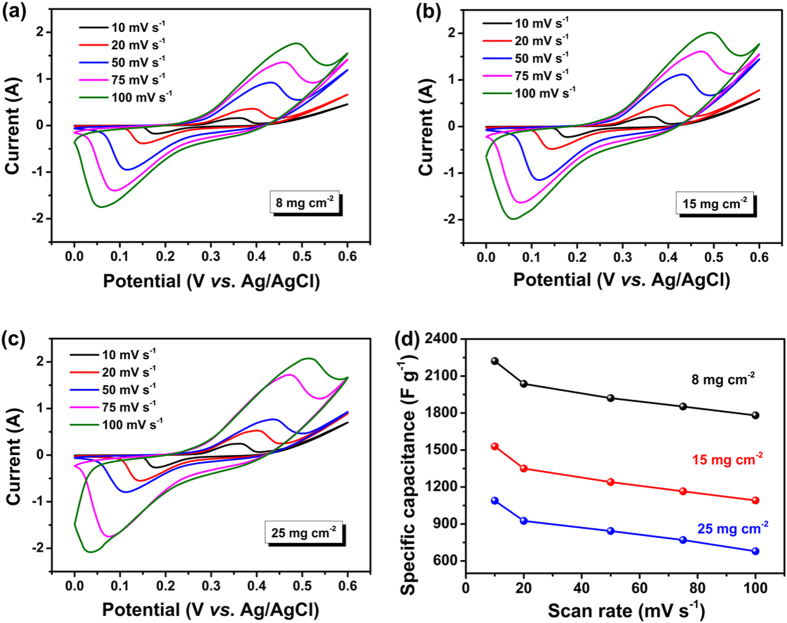
CV curves of the CuS/CNT electrodes with varying mass loadings of (**a**) 8, (**b**) 15 and (**c**) 25 mg cm^−2^ obtained at different scan rates; and (**d**) variations of specific capacitance of the CuS/CNT electrodes as a function of scan rate.

Figure 7a–c present the CV curves of the CuS/CNT electrodes with different mass loadings measured at different scan rates. The non-rectangular shape of the CV curves for all mass loadings is not obviously changed by increasing the scan rate, and the peak current rises with the scan rate, suggesting that the architecture of CuS/CNT composites is good for fast redox reactions. Also, it is apparent that the redox peaks shift to lower and higher voltages in higher scan rates, respectively. In general, ion diffusion is confined only to the surfaces of the electrode material at a high scan rate^40^,^41^. The area under the CV curves increased gradually with increasing scan rate for all mass loadings studied, implying an ideal capacitive behavior^42^.

The specific capacitance of the electrodes calculated according to Eqn (2) is designed as a function of scan rate, as shown in Fig. 7d. It is obvious that the lower were the electrode mass loading and scan rate, the higher was the specific capacitance. The maximum specific capacitances achieved were 2221, 1524, and 1084 F g^−1^ for 8, 15, and 25 mg cm^−2^ mass loadings, respectively, when measured at 10 mV s^−1^ scan rate. Even at a high scan rate of 100 mV s^−1^, the specific capacitance remained a remarkable value of 1770 F g^−1^ for 8 mg cm^−2^. There was a significant synergy arising from the incorporation of CNT to form 3D conducting networks between the whole active materials. The CNT fully integrated into the unique hierarchical structure not only enhanced the electronic conductivity of the electrode, but also served as a buffer to release the volumetric strains of the CuS particles during the charge/discharge process. In addition, the thin CuS nanosheet gave rise to a large surface area and porosity as well as a short diffusion path for ion transport, all of which positively contributed to the enhanced specific capacitance and cyclic stability of the composite electrodes^43^.

Typical charge/discharge profiles of the CuS/CNT electrode with a loading of 8 mg cm^−2^ measured at different various densities are given in Figure S2a. It can be seen that all of the curves are very symmetric and show ideal capacitive behavior. Prominently, a small drop in potential is caused by the low internal resistance of electrode. The SCs of the electrodes calculated according to eqn (3) are plotted as a function of current density, as shown in Figure S2b. The electrode with 8 mg cm^−2^ delivered the highest SCs among the three at all current densities studied: e.g. 2204 and 1708 F g^−1^ at 10 and 160 mA cm^−2^, respectively. These values are about 1.5 times those of the electrode with 15 mg cm^−2^ or 2 times those of the electrode with 25 mg cm^−2^ at the same discharge current densities. The C_s_ decreased with increasing specific current density similar to that observed in the CV tests. Correspondence between specific capacitances and capacities at different current densities is shown in Figure S3. As can be seen, there was an excellent capacity value of 368 mA h g^−1^ at 10 mA cm^−2^.

**Figure 8:**
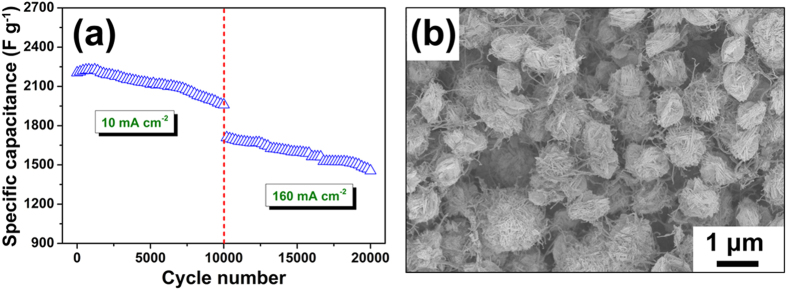
(**a**) Cycling performance of the CuS/CNT electrode with a loading of 8 mg cm^−2^ measured by charge-discharge; (**b**) SEM image of the CuS/CNT electrode with a loading of 8 mg cm^−2^ after 20000 cycles.

The stability of the CuS/CNT electrode with 8 mg cm^−2^ was measured by galvanostatic charge-discharge (Fig. 8a). The C_s_ was 1960 F g^−1^ at a current density of 10 mA cm^−2^ after 10000 cycles (89% retention of the initial value of 2204 F g^−1^). Subsequently, the current density was extended to 160 mA cm^−2^, the C_s_ remained 1236 F g^−1^ (85% of 1454F g^−1^) after another 10000 cycle. In addition, SEM image (Fig. 8b) of the CuS/CNT electrode after cycling test exhibits that CNT were still inserted between the CuS nanosheets, which demonstrates the structural stability of these microspheres as an electrode for supercapacitors. Such an excellent cycling stability can be attributed to the hierarchical sphere-shaped architecture, the well-separated nanosheets were exposed to the electrolyte, offering sufficient diffusion channels. Meanwhile, void spaces between the neighboring nanosheets play the role of ion buffering reservoirs, ensured sufficient redox reactions to take place at high current densities.

**Figure 9:**
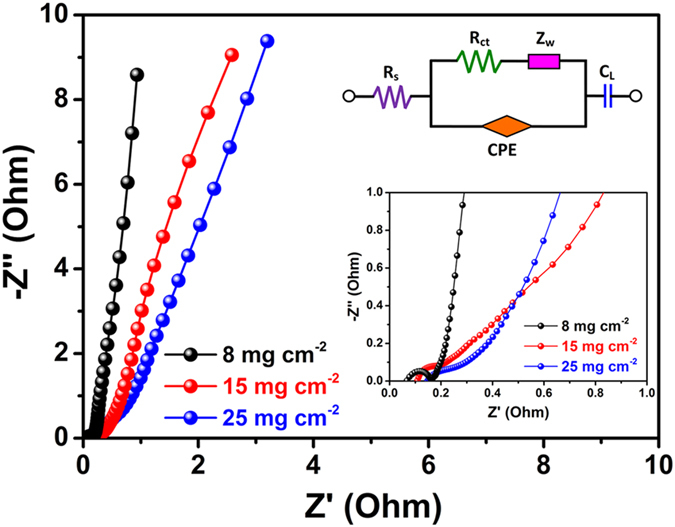
Electrochemical impedance spectra (EIS) of the CuS/CNT electrodes with different mass loadings (insets: corresponding equivalent circuit and magnified plot).

Nyquist plots of the EIS spectra of CuS/CNT-based electrode materials are shown in Fig. 9. The inset of Fig. 9 gives an equivalent circuit used for fitting the EIS curves. From the intercepts of the high-frequency semicircle on the real axis, the bulk solution resistance R_s_ and charge-transfer resistance R_ct_ can be calculated^44^. According to the data (Table S1), both the R_s_ and R_ct_ values of the CuS/CNT electrode with a loading of 8 mg cm^−2^ were lower than those of the two other electrodes. As is known to all, the charge-transfer resistance is an important parameter, which restricts the specific power of a supercapacitor^45^. Hence, CuS/CNT electrode with a loading of 8 mg cm^−2^ has a better practical value. In view of the very steep slopes of the electrodes, it is thought that the thin nanosheet structure of the individual CuS particles ameliorated the ion transportation in the aqueous electrolyte.

## Conclusion

In summary, we developed a simple PVP-assisted reflux method for large-scale synthesis of novel 3D hierarchical CuS and CuS/CNT microspheres. The porous sphere-like structure could remain the same and inserted thoroughly by CNT, even after 20000-cycle charge/discharge tests. This composite structure could achieve both large specific area and good architectural stability as electrode materials for a supercapacitor. The synergistic effect caused by the incorporation of CNT and the ultra-thin CuS nanosheets gives the CuS/CNT composites large specific capacitance and remarkable cycling stability. Accordingly, these sphere-like structures of CuS interlaced with CNT may possess great prospect for advanced electrochemical energy storage applications.

## Methods

### Preparation of 3D hierarchical CuS microspheres

All the chemicals were of analytical grade reagent and used without further purification. In a typical synthesis process, 1 g of Polyvinyl Pyrrolidone (PVP) was dissolved in 40 mL deionised (DI) water under magnetic stirring, followed by the addition of 2 mmol Cu(NO_3_)_2_·3H_2_O to form a blue solution. 30 mL ethylene glycol (EG) and 4 mmol thiourea (THU) were added to the mixture; and after stirring for 1 h the dark blue solution was transferred to a three-necked flask. The mixture was then refluxed while vigorously stirring at 160 ^o^C for 2 h, where the solution turned black. Subsequently, the solution was allowed to cool to room temperature naturally. The black suspension and precipitates were separated by centrifugation, which washed with absolute ethanol for several times. And then collected and dried overnight at 80 ^o^C.

### Preparation of 3D hierarchical CuS/CNT composites

For the synthesis of CuS/CNT composites, multi-walled CNT (MWCNT were purchased from Shenzhen Nanotech Port Co., Ltd. China, 40–60 nm in outer diameter and 0.5–2 μm in length) pretreated by refluxing in boiling concentrated nitric acid for 6 h to remove any impurity as well as to oxidize the opened end of the tubes. 15 mg of the acid-functionalized CNT was ultrasonically dispersed in 5 mL of dimethyl formamide (DMF) for 2 h, and the mixture was added into the above dark blue solution. The rest of the procedure was essentially the same as the neat CuS.

### Materials characterizations

The phase structures of the materials were characterized by X-ray diffraction (XRD) analysis on a D8 Focus (Bruker, Germany) automated X-ray diffractometer system with Cu-Kα radiation (λ = 1.5418 Å). The Raman scattering measurement was carried out on a laser Raman spectrometer (Renishaw, England) at room temperature. Their morphologies and structures were characterized by field emission scanning electron microscopy (FSEM, JEOL S-4800) and transmission electron microscopy (TEM, JEOL JEM-2010). The content of CuS in the composite was determined using thermogravimetric analysis (TGA, PerkinElmer) at a heating rate of 10 ^o^C min^−1^ in flowing air. Nitrogen adsorption/desorption isotherms were determined at 77 K on a Micrometritics ASAP 2020 analyzer. The pore size distributions were calculated based on a density functional theory (DFT) method using the nitrogen adsorption data and assuming a slit pore model.

### SC performance measurements

The pseudocapacitive performance of the electrodes made from the CuS-CNT composites were investigated on an electrochemical workstation, CHI660E (Chenhua, P. R. China) using a three-electrode system. The working electrode was prepared by mixing the active material, carbon black and polyvinylidene fluoride (PVDF) binder (10% solution in N-methyl-2-pyrollidone) with a mass ratio of 90:5:5. A 5% solution of the mixture in isopropanol was sprayed on Ni foam as the current collector. The prepared electrode was dried at 60 ^o^C overnight. A Pt plate was used as the auxiliary electrode and Ag/AgCl as the reference electrode, with 2 M KOH solution as the electrolyte at room temperature. The cyclic voltammetry (CV) analysis was performed between 0 and 0.6 V vs. Ag/AgCl at scan rates ranging from 10 to 100 mV s^−1^. The galvanostatic charge/discharge test was conducted in a stable potential window at different current densities of 10–160 mA cm^−2^. The electrochemical impedance spectroscopy (EIS) was performed at an AC voltage of 5 mV in the frequency range from 0.01 Hz to 100 kHz. The nominal area of the CuS/CNT/Ni foam electrode immersed in the electrolyte was controlled at around 1 cm × 1 cm. The specific capacitances (C_s_) of the electrodes were estimated from the cathodic or anodic part of the CV curves according to the following equation:


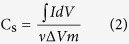


where *I* (A) is the response current, *ν* (V s^−1^) is the potential scan rate, Δ*V* (V) is the potential window, and *m* (g) is the mass of the active electrode material. Meanwhile, the C_s_ was also calculated from the galvanostatic discharge curves according to the following equation:


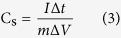


where *I* (A), Δ*t* (s), Δ*V* (V) and *m* (g) are the specific capacitance, the constant discharge current, the total discharge time, the potential window and the mass of the electroactive materials, respectively.

## Additional Information

**How to cite this article**: Lu, Y. *et al.* Hierarchical, porous CuS microspheres integrated with carbon nanotubes for high-performance supercapacitors. *Sci. Rep.* **5**, 16584; doi: 10.1038/srep16584 (2015).

## Supplementary Material

Supplementary Information
