# Supplementary material for: Hierarchical, porous CuS microspheres integrated with carbon nanotubes for high-performance supercapacitors
Source: Sci Rep. 2015 Nov 16;5:16584. doi: 10.1038/srep16584 (PMC4645118; doi:10.1038/srep16584)
Supplement: Supplementary Information [file srep16584-s1.docx]

**Supporting information**

**Hierarchical, porous CuS microspheres integrated with carbon nanotubes for high-performance supercapacitors**

Yang Lu^1,2^, Xianming Liu^3^, Weixiao Wang^1^, Jinbing Cheng^1^, Hailong Yan^1^, Chengchun Tang^2^, Jang-Kyo Kim^4^ & Yongsong Luo^1^

^1^Key Laboratory of Advanced Micro/Nano Functional Materials, School of Physics and Electronic Engineering, Xinyang Normal University, Xinyang, P. R. China;

^2^School of Material Science and Engineering, Hebei University of Technology, Tianjin, P. R. China;

^3^College of Chemistry and Chemical Engineering, Luoyang Normal University, Luoyang, P. R. China;

^4^Department of Mechanical and Aerospace Engineering, The Hong Kong University of Science and Technology, Clear Water Bay, Kowloon, Hong Kong, P. R. China

Correspondence: Professor Yongsong Luo, Key Laboratory of Advanced Micro/Nano Functional Materials, School of Physics and Electronic Engineering, Xinyang Normal University, Xinyang, P. R. China, E-mail address: [ysluo@xynu.edu.cn](mailto:ysluo@xynu.edu.cn)

Professor Chengchun Tang, School of Material Science and Engineering, Hebei University of Technology, Tianjin, P. R. China, E-mail address: [tangcc@hebut.edu.cn](mailto:tangcc@hebut.edu.cn)


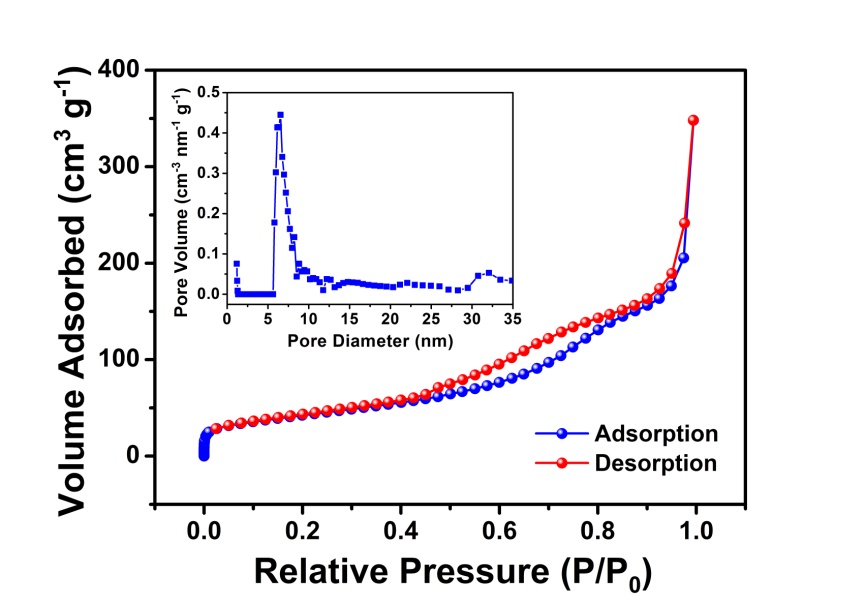


**Figure S1** Nitrogen adsorption/desorption isotherms of CuS/CNT composites. Inset presents the corresponding pore size distribution curve.


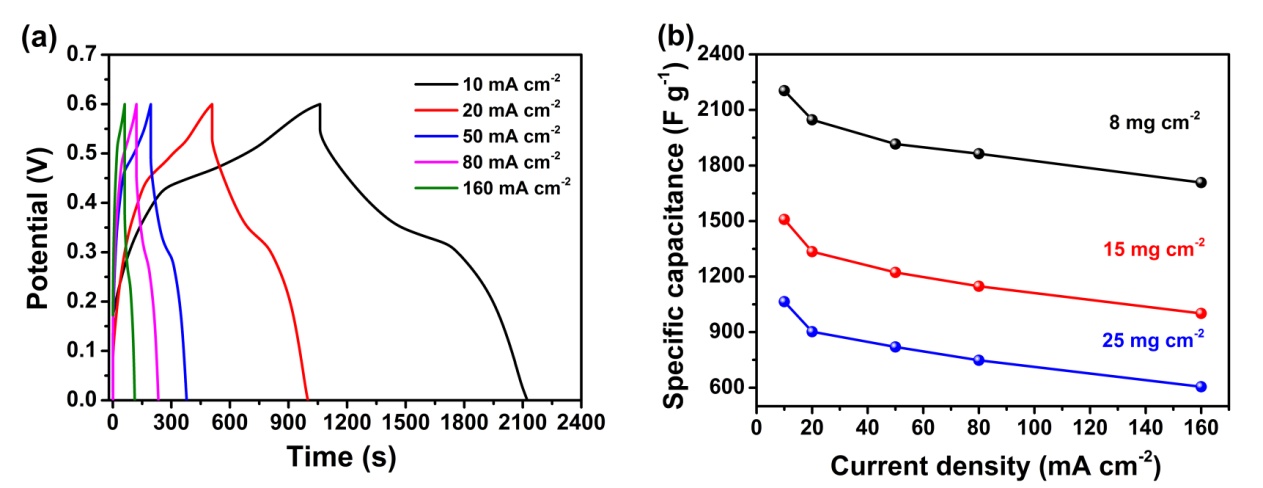


**Figure S2** (a) Constant current charge/discharge curves of the CuS/CNT electrode with 8 mg cm^-2^ mass loading measured at different current densities; and (b) variations of specific capacitance of the CuS/CNT electrodes as a function of current density.


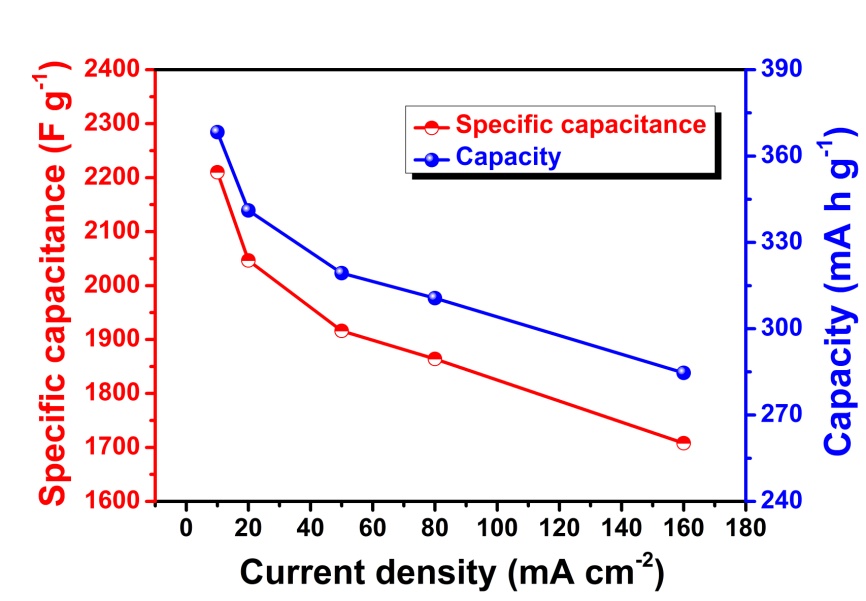


**Figure S3** Specific capacitances and capacities of the CuS/CNT electrode with a loading of 8 mg cm^-2^ at different current densities.

**Table S1** Charge transfer resistance (R_ct_) and internal resistance (R_s_) of the CuS/CNT-based electrode materials.

| Sample | R_ct_ (Ω) | R_s_ (Ω) |
| --- | --- | --- |
| 8 mg cm^-2^ | 0.1 | 0.06 |
| 15 mg cm^-2^ | 0.14 | 0.11 |
| 25 mg cm^-2^ | 0.18 | 0.15 |
